# Case report: Multisystemic smooth muscle dysfunction syndrome: a rare genetic cause of infantile interstitial lung disease

**DOI:** 10.3389/fphar.2024.1510969

**Published:** 2025-01-09

**Authors:** Qianying Li, Lidan Cui, Jun Su, Yuelin Shen

**Affiliations:** ^1^ Pediatric Intensive Care Unit, Children’s Hospital Affiliated to Zhengzhou University, Henan Children’s Hospital, Zhengzhou Children’s Hospital, Zhengzhou, China; ^2^ Respiratory Department II, National Clinical Research Center for Respiratory Diseases, Beijing Children’s Hospital, Capital Medical University, National Center for Children’s Health, Beijing, China; ^3^ Respiratory Department, Children’s Hospital Affiliated to Zhengzhou University, Henan Children’s Hospital, Zhengzhou Children’s Hospital, Zhengzhou, China

**Keywords:** interstitial lung disease, multisystemic smooth muscle dysfunction syndrome, congenital mydriasis, infant, case report

## Abstract

Multisystemic smooth muscle dysfunction syndrome (MSMDS) is an autosomal dominant disorder caused by mutations in the *ACTA2* gene, resulting in variable clinical manifestation and multi-organ dysfunction. Interstitial lung disease (ILD) is a rare phenotype of this condition. We describe a rare infant case of an 8-month-old boy who presented with progressively worsening dyspnea, along with intermittent episodes of respiratory distress and cyanosis since birth. A chest CT scan revealed typical signs of ILD. Additionally, the patient exhibited congenital mydriasis, aortic coarctation, PDA, and pulmonary hypertension. Whole-exome sequencing identified a *de novo* variant c.536G > A (p.Arg179His) in the *ACTA2* gene. These findings confirmed the diagnosis of MSMDS. Despite intensive hospital-based pulmonary care and optimized therapy, the child passed away due to sudden cardiac and respiratory arrest on the 12th day of hospitalization. This case underscores the importance of considering MSMDS in the differential diagnosis of infantile ILD.

## Introduction

Multisystemic smooth muscle dysfunction syndrome (MSMDS) is an autosomal dominant disorder caused by mutations in the *ACTA2* gene (Milewicz et al., 2010), which encodes vascular smooth muscle α-actin (SM α-actin). Mutations in SM α-actin may profoundly affect its interaction with myosin, other actin protomers within the filament, actin-binding proteins that regulate the monomer-polymer equilibrium within the cell, and other binding partners that impact cellular phenotype (Lu et al., 2016). The clinical manifestations of MSMDS are diverse, involving multiple organ systems such as the ocular, cardiovascular, neurological, gastrointestinal, genitourinary and respiratory systems (Lupo et al., 2023; Prabhu et al., 2017).

Interstitial lung disease (ILD) consists of a group of pulmonary disorders characterized by inflammation and/or fibrosis of the lung parenchyma associated with progressive dyspnea that frequently results in end-stage respiratory failure (Maher, 2024). ILD is an uncommon phenotype of MSMDS.

In this study, we reported a case of MSMDS in an 8-month-old Chinese boy who presented with infantile ILD as the initial and predominant manifestation. This case highlights the importance of considering MSMDS in the differential diagnosis of infantile ILD, given the broad spectrum of clinical manifestations and the potential for significant respiratory involvement.

## Case presentation

**FIGURE 1:**
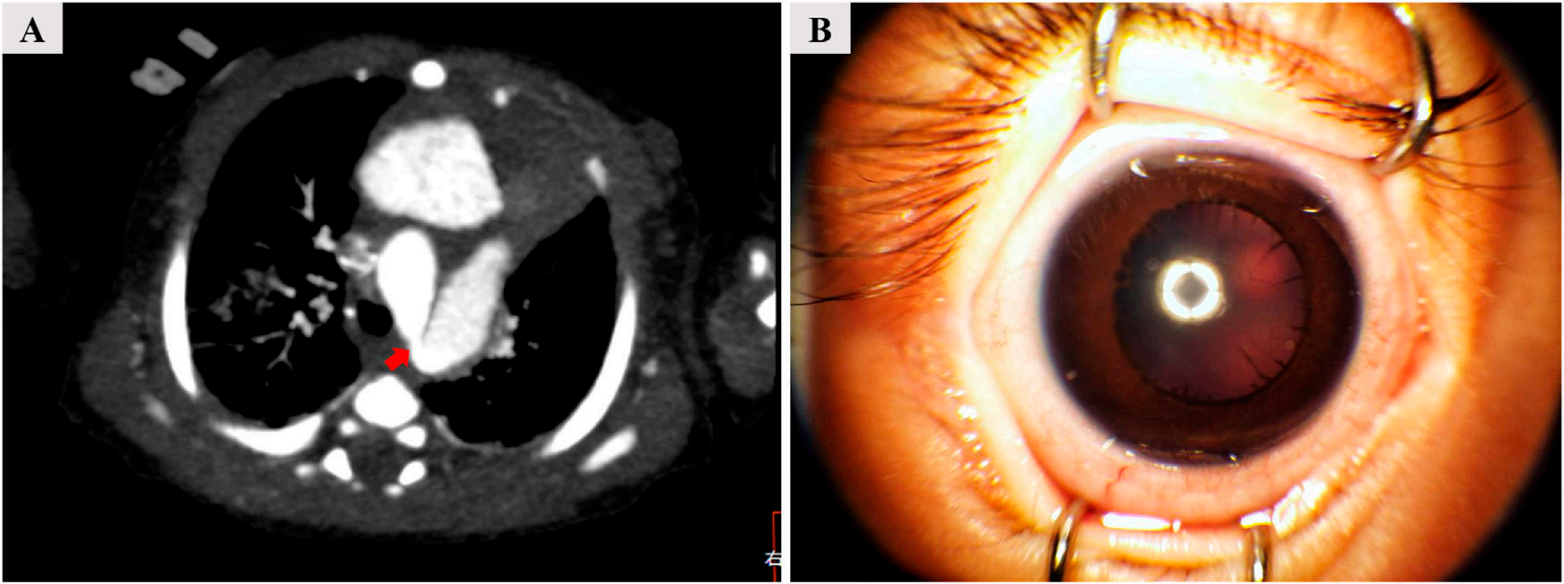
Examination images confirm severe aortic coarctation on the first day of life and congenital mydriasis at 8 months. **(A)** Cardiac computed tomography angiography revealing aortic coarctation, with the narrowest point measuring 2.3 mm (arrow). **(B)** Ophthalmologic examination revealing bilaterally fixed pupils dilated to 5 mm, with an absence of both direct and consensual pupillary light reflexes.

An 8-month-old boy presented to the emergency department of the Children’s Hospital Affiliated to Zhengzhou University with a 3-day history of dyspnea (shortness of breath) and cyanosis. He had exhibited progressively worsening dyspnea, along with intermittent episodes of respiratory distress and cyanosis since birth. This was his fifth hospitalization due to similar symptoms. His family history was unremarkable. During his mother’s pregnancy, a fetal ultrasound at 30 weeks of gestation detected aortic coarctation. He was born at full term. On the first day of life, congenital mydriasis was noted. A cardiac computed tomography (CT) angiogram confirmed severe aortic coarctation, revealing a narrowest point of 2.3 mm (Figure 1A). An echocardiography showed pulmonary hypertension (tricuspid regurgitation pressure gradient 62 mmHg) with patent ductus arteriosus (PDA). On the eighth day of life, the patient underwent cardiac surgery and was subsequently treated with spironolactone, furosemide, and bosentan. Despite these interventions, his dyspnea gradually worsened.

Upon physical examination during this admission, the patient had a temperature of 36.4°C (97.5°F), blood pressure of 84/45 mmHg, heart rate of 160 beats per minute, respiratory rate of 51 breaths per minute, and an oxygen saturation of 80% on room air. Additionally, cyanosis of the lips was observed. Furthermore, ophthalmologic examination revealed bilaterally fixed pupils dilated to 5 mm, with an absence of both direct and consensual pupillary light reflexes (Figure 1B). Lung auscultation revealed bilateral crackles in the lower lobes. Developmental history indicated that all milestones were at the level of a 5-month-old.

**FIGURE 2:**
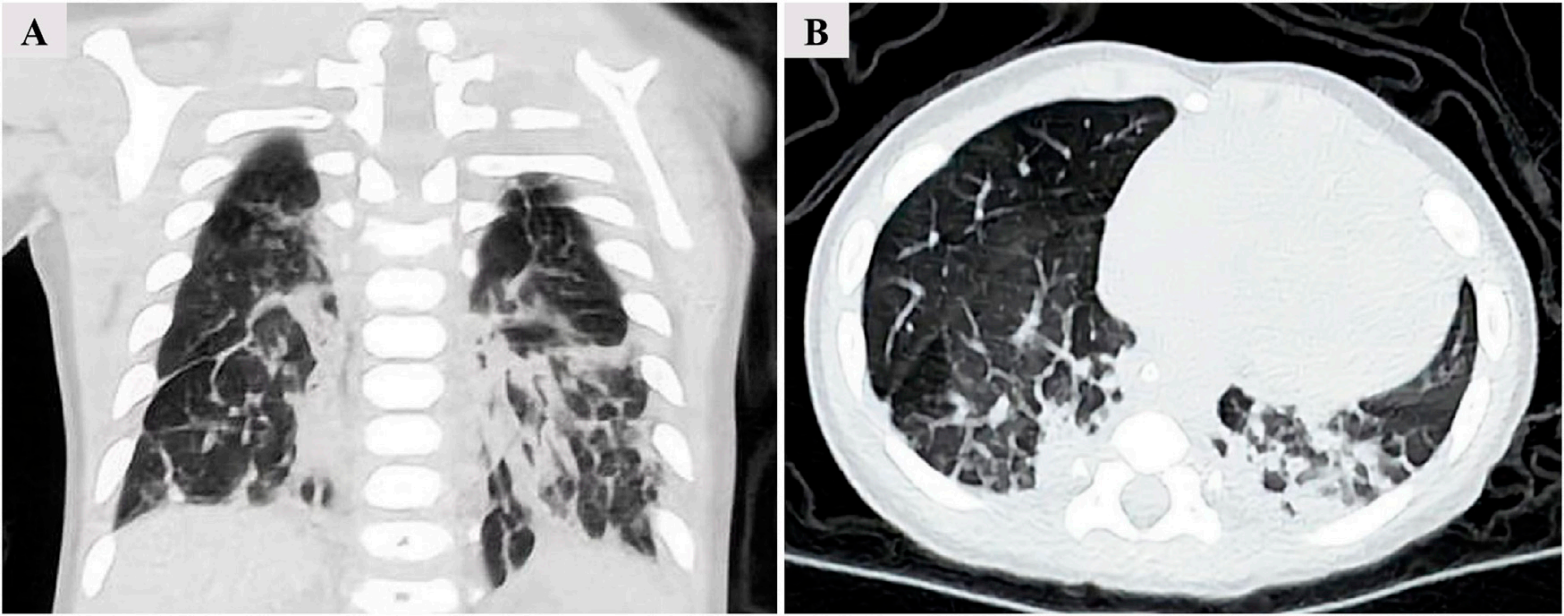
Chest CT demonstrating interstitial infiltrates in both lower lungs, accompanied by interlobular septal and pleural thickening. **(A)** Coronal view. **(B)** Transverse view.

Upon admission, arterial blood gas analysis revealed a pH of 7.396, a PaCO_2_ of 36.5 mmHg, and a PaO_2_ of 65.4 mmHg, while the patient was receiving nasal cannula oxygen therapy at a flow rate of 2L/min. A chest CT demonstrated interstitial infiltrates in both lower lungs, accompanied by interlobular septal and pleural thickening (Figures 2A, B). Chromosomal analysis was normal. To further clarify the etiology of infantile ILD, the patient and his parents underwent whole-exome sequencing (WES).

**FIGURE 3:**
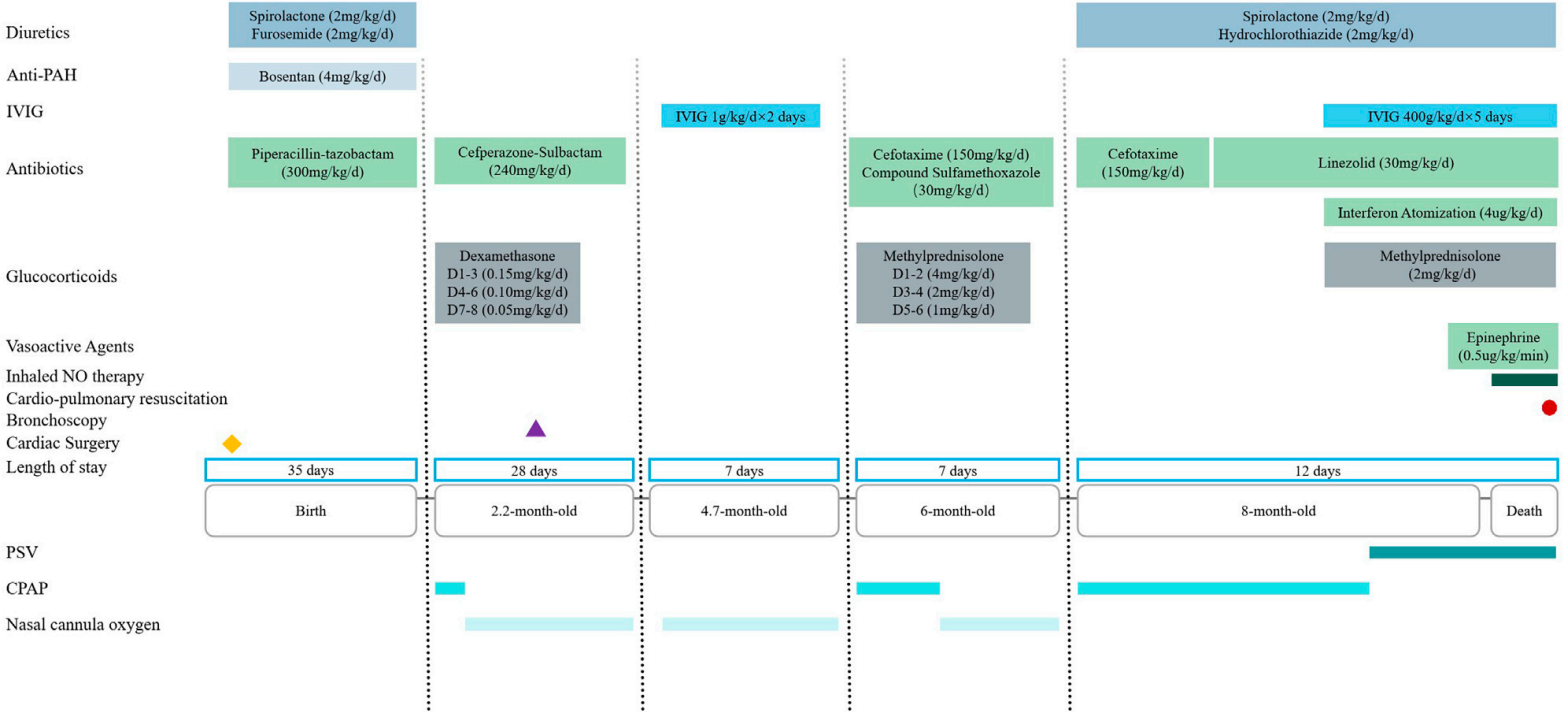
Timeline of medical history from birth to death in a 8-month-old boy with MSMDS. Abbreviation: PAH = Pulmonary hypertension; IVIG: Intravenous immunoglobulin; NO = Nitric oxide; PSV = Pressure support ventilation; CPAP = Continuous positive airway pressure.

The patient received intensive care support. Upon admission, since nasal cannula oxygen could not improve hypoxemia, continuous positive airway pressure was initiated. Intravenous cefotaxime (150 mg/kg/day) was administered for empirical antibacterial therapy. On the fourth day of hospitalization, the patient developed a high-spiking fever. A repeat complete blood count showed an elevated white blood cell count of 20 × 10^9^/L, up from the admission count of 14 × 10^9^/L. Sputum culture revealed *Staphylococcus aureus*, prompting the cessation of cefotaxime and initiation of linezolid (30 mg/kg/day) for antibacterial treatment. On the seventh day of hospitalization, the patient experienced respiratory distress and worsening hypoxemia. Sputum tested positive for respiratory syncytial virus DNA, leading to the addition of interferon nebulization (4 μg/kg/day), intravenous methylprednisolone (2 mg/kg/day), and intravenous immunoglobulin (400 mg/kg/day for 5 days) therapy. However, these treatments did not yield any improvement in symptoms. On the ninth day of hospitalization, endotracheal intubation and invasive mechanical ventilation were administered. Despite continuous mechanical ventilation, oxygenation could not be maintained, resulting in persistent hypoxemia. On the 11th day, inhaled nitric oxide therapy was initiated. On the 12th day of hospitalization, the patient suffered sudden cardiac and respiratory arrest. Cardiopulmonary resuscitation and epinephrine (0.5 μg/kg/min) were administered, but the patient ultimately passed away (Figure 3).

**FIGURE 4:**
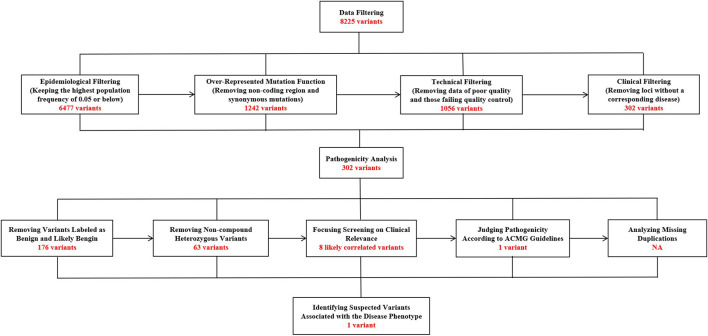
The specific screening process of whole-exome sequencing in this 8-month-old patient with MSMDS.

Two weeks after the patient’s death, WES results were finally available, identifiing a heterozygous *de novo* variant c.536G > A (p.Arg179His) in exon six of the *ACTA2* gene (Figure 4), which eventually confirmed the diagnosis of MSMDS.

## Discussion

In this study, the patient exhibited infantile ILD as the predominant manifestation, accompanied by multi-system involvements such as congenital mydriasis, aortic coarctation, PDA, and pulmonary hypertension. WES identified a *de novo* variant in the *ACTA2* gene, confirming a diagnosis of MSMDS. This condition was first reported in 2010 (Milewicz et al., 2010), to date, there have been 90 globally documented cases (including the current patient) (Lupo et al., 2023; Prabhu et al., 2017; Chen et al., 2019; Houska et al., 2022; Yang et al., 2021; Amans et al., 2014; Taubenslag et al., 2019; Lino Cardenas et al., 2023; Zhou et al., 2017; Roylance et al., 2023; She et al., 2021; Mc Glacken-Byrne et al., 2020; Ng et al., 2024; de Grazia et al., 2017; Moller et al., 2012; Logeswaran et al., 2017; Kanamori et al., 2021; Moreno et al., 2016; Munot et al., 2012; Meuwissen et al., 2013; Ardhanari et al., 2020; Richer et al., 2012; Roulez et al., 2014; Yetman et al., 2015; Moosa et al., 2013; Amans et al., 2013; Brodsky et al., 2014; Micke et al., 2023; Zhou et al., 2020; Kaw et al., 2022), with 80.0% of these reports originating from Europe and the United States. This condition is rare in East Asia, with only seven reported cases in China (Chen et al., 2019; Yang et al., 2021; Zhou et al., 2017; She et al., 2021; Ng et al., 2024; Zhou et al., 2020) and 1 case in Japan (Kanamori et al., 2021). Among the reported patients, the age of onset varies widely, ranging from fetal stages to adulthood (Yetman et al., 2015; Micke et al., 2023; Zhou et al., 2020; Regalado et al., 2018), and 66.7% of the cases were female. Clinical manifestations of MSMDS are variable and include ocular (congenital mydriasis), cardiovascular (e.g., PDA, aortic aneurysm, pulmonary hypertension), neurological (e.g., cerebral infarction, hemiplegia, motor/mental delay), gastrointestinal (intestinal malrotation, intestinal dyskinesia), genitourinary (hypotonic bladder, hydronephrosis) and pulmonary (recurrent respiratory infection and ILD) multi-organ involvement (Yetman et al., 2015; Regalado et al., 2018). Among these, congenital mydriasis and PDA are the most common, affecting approximately 92.6% and 91.6% of patients, respectively. Conversely, ILD is relatively rare, accounting for 13.3%.

ILD comprises a large group of diseases that involve the lung interstitium and parenchyma. These diseases are characterized by complex and diverse pathological changes, a wide range of imaging findings, and various etiologies. Among these, ILD occurring in infancy (also known as infantile ILD) is associated with congenital malformations, genetic mutations, or chronic lung damage resulting from premature birth or other congenital anomalies (Kitazawa and Kure, 2015). In this case, the infant patient presented with progressively worsening dyspnea, along with intermittent episodes of respiratory distress and cyanosis since birth. A chest CT scan revealed bilateral interstitial infiltrates, confirming the diagnosis of infantile ILD. The patient was born at full term, ruling out bronchopulmonary dysplasia. Chromosomal analysis yielded normal results, excluding chromosomal aberrations (such as trisomy 21) as a cause of impaired lung growth. Furthermore, WES ruled out genetic surfactant dysfunctions or alveolar-capillary dysplasia with pulmonary vein misalignment. Based on the multisystem involvement observed in this patient and the presence of a *de novo* variant in the *ACTA2* gene, we conclude that infantile ILD in this case was caused by MSMDS. This case underscores the importance of considering MSMDS in the differential diagnosis of infantile ILD.

Among the 90 reported cases of MSMDS, 12 (13.3%) involved ILD (Milewicz et al., 2010; Lupo et al., 2023; Prabhu et al., 2017; Chen et al., 2019; Houska et al., 2022; Zhou et al., 2017; Richer et al., 2012; Yetman et al., 2015). The chest imaging of these ILD cases showed inhomogeneous lung transparency, cystic changes, interlobular septal thickening, and fibrotic bands. Only 5 cases underwent lung biopsy, revealing pathological features such as simplified alveolar structures, glycogen dissolution in the lung interstitium, enlarged alveolar spaces, intimal hyperplasia with early fibrosis, and alveolar dysplasia (Milewicz et al., 2010; Prabhu et al., 2017; Houska et al., 2022; Richer et al., 2012). All 12 cases of ILD manifested in infancy, with 11 cases associated with the p.Arg179His variant and 1 case with the p.Arg179Cys variant. Prognosis for most patients remains unclear. One patient was followed up for 1.5 years and is currently stable (Chen et al., 2019); another patient was followed up for 6 months and requires oxygen therapy (Richer et al., 2012). Two patients passed away, one of which was our case, and the other died at 11 months of age (Yetman et al., 2015).

Among the total 90 MSMDS patients, genotypes were clearly described in 85 cases. According to the Human Gene Mutation Database (HGMD, April 2024), there are 120 documented *ACTA2* variants, including 108 missense/nonsense, five splice site, and seven microdeletions. Notably, the p.Arg179His variant is the most prevalent (Munot et al., 2012; Kaw et al., 2022; Regalado et al., 2018), accounting for 63.5% of cases. It is described as pathogenic in the ClinVar database and is assessed as disease-causing by scholars in the HGMD.This variant has the potential to affect actin structure and function in both the contractile domain of the cell and the more dynamic cytoskeletal pool of actin, both of which are necessary for contraction (Lu et al., 2016). It represents the most severe end of the *ACTA2* variant spectrum, characterized by its very early onset and highly penetrant vascular diseases in affected patients. Less common variants include p.Arg179Cys, p.Arg179Leu, p.Arg179Ser, p.Arg179Gly, p.Asn117Lys, and p.Arg189His (Logeswaran et al., 2017; Kaw et al., 2022).

As of the current date, no definitive standard of care has been established for MSMDS. Management of this condition is predominantly symptomatic. For severe cardiovascular anomalies, surgical interventions such as arterial duct ligation and aortic coarctation resection may be performed. Diuretics and sildenafil/bosentan can be administered to reduce cardiac load and alleviate pulmonary hypertension, respectively. For ILD, oxygen therapy, antimicrobial, and anti-inflammatory therapy can be administered. For patients with central nervous system involvement, medication can be used to control epileptic seizures. Vascular reconstruction can be considered for patients with occlusive cerebrovascular disease; however, the risk of ischemic stroke post-reconstruction remains high and is still a matter of debate. Given its multi-systemic involvement, the prognosis for this syndrome is unfavorable. Unfortunately, most reported cases lack long-term follow-up. Among all patients with the p.Arg179His variant, 10 cases (18.5%) had already passed away at the time of reporting, with ages at death ranging from 0.5 to 32.7 years (Mc Glacken-Byrne et al., 2020; Yetman et al., 2015; Regalado et al., 2018). Six patients (11.1%) are currently under follow-up. Regalado et al. reported one male patient who underwent congenital heart disease repair at the age of 2, experienced aortic dissection at 17, and had an aortic aneurysm repair at 18. He was genetically diagnosed with MSMDS at 36.6 years and was last followed up at 37.4 years, making him the oldest surviving MSMDS patient to date (Regalado et al., 2018). Additionally, 38 patients (70.4%) have no follow-up or prognosis information available. For cases with non-p.Arg179His variants, there have been five reported deaths (de Grazia et al., 2017; Moller et al., 2012; Meuwissen et al., 2013; Kaw et al., 2022; Regalado et al., 2018). This includes one case associated with the p. Arg179Gly variant, with an age of death at 27 days (Kaw et al., 2022). Three cases involved the p.Arg179Cys variant, with ages at death of 2.5 months, 3 years, and 30.4 years, respectively (de Grazia et al., 2017; Meuwissen et al., 2013; Regalado et al., 2018). One case was a somatic mosaic for a novel p.Asn117Lys transversion in the *ACTA2* gene, diagnosed at 62 years and passed away at 66 years (Mc Glacken-Byrne et al., 2020).

The limitation of the present study is that, despite this patient being hospitalized five times for respiratory distress and cyanosis, and exhibiting typical multi-system involvements, MSMDS was only considered during the final hospitalization. Due to the time-consuming of WES testing, he was only definitively diagnosed 2 weeks after his death. Consequently, he did not receive a correct diagnosis or long-term follow-up during his lifetime. We hope that this case report of this rare disease will draw attention to infantile ILD due to MSMDS.

In conclusion, this case underscores the importance of considering MSMDS in the differential diagnosis of infantile ILD. Although ILD is an infrequent manifestation of MSMDS, the presence of multi-system involvement and the identification of *ACTA2* gene variants are essential for diagnosis.

## Data Availability

The original contributions presented in the study are included in the article/supplementary material, further inquiries can be directed to the corresponding author.
